# Impact of maternal antenatal nutrition and infection treatment interventions on Longitudinal Infant Development and Growth in rural Ethiopia: protocol of the LIDG child follow-up study

**DOI:** 10.1136/bmjpo-2024-002840

**Published:** 2024-12-24

**Authors:** Firehiwot Workneh, Theresa I Chin, Kalkidan Yibeltal, Nebiyou Fasil, Krysten North, Sarah K G Jensen, Workagegnhu Tarekegn Kidane, Mulatu Melese, Sitota Tsegaye, Yoseph Yemane Berhane, Unmesha Roy Paladhi, Betelhem Haimanot Abate, Atsede Teklehaimanot, Tizita Lemma Melka, Stephen Pihl, Winko W An, Fred Van Dyk, Luke C Mullany, Lian V Folger, Sara Cherkerzian, Sonya V Troller-Renfree, Moriah E Thomason, Maria Andersson, Terrie Inder, Charles A Nelson, P Ellen Grant, Parul Christian, Alemayehu Worku, Yemane Berhane, Anne CC Lee

**Affiliations:** 1 Department of Epidemiology and Biostatistics, Addis Continental Institute of Public Health, Addis Ababa, Ethiopia; 2 Department of Pediatrics, Brigham and Women's Hospital, Boston, Massachusetts, USA; 3 Harvard Medical School, Boston, Massachusetts, USA; 4 Department of Pediatrics, Warren Alpert Medical School of Brown University, Providence, Rhode Island, USA; 5 Department of Reproductive Health and Population, Addis Continental Institute of Public Health, Addis Ababa, Ethiopia; 6 Department of Global Health and Health Policy, Addis Continental Institute of Public Health, Addis Ababa, Ethiopia; 7 Boston Children's Hospital, Boston, Massachusetts, USA; 8 Harvard T H Chan School of Public Health, Boston, Massachusetts, USA; 9 Department of Nutrition and Behavioural Science, Addis Continental Institute of Public Health, Addis Ababa, Ethiopia; 10 Amhara Public Health Institute, Bahir Dar, Ethiopia; 11 Addis Continental Institute of Public Health, Bahir Dar, Ethiopia; 12 Addis Ababa University College of Health Science, Tikur Anbessa Specialized Hospital, Addis Ababa, Ethiopia; 13 Department of Psychology, Bahir Dar University, Bahir Dar, Ethiopia; 14 Department of International Health, Johns Hopkins Bloomberg School of Public Health, Baltimore, Maryland, USA; 15 Department of Human Development, Teachers College at Columbia University, New York, New York, USA; 16 Department of Child and Adolescent Psychiatry, New York University Grossman School of Medicine, New York, New York, USA; 17 Nutrition Research Unit, Children's Research Centre, University Children's Hospital Zurich - Eleonore Foundation, Zurich, Switzerland; 18 Center for Neonatal Research, Children's Hospital of Orange County, Orange, California, USA

**Keywords:** Nutrition, Child Health, Infant, Low and Middle Income Countries

## Abstract

**ABSTRACT:**

**Introduction:**

Maternal undernutrition and inflammation in utero may significantly impact the neurodevelopmental potential of offspring. However, few studies have investigated the effects of pregnancy interventions on long-term child growth and development. This study will examine the effects of prenatal nutrition and infection management interventions on long-term growth and neurodevelopmental outcomes of offspring.

**Methods:**

The Enhancing Nutrition and Antenatal Infection Treatment (‘ENAT’) study (ISRCTN15116516) was a pragmatic, open-label, 2×2 factorial, randomised clinical effectiveness study implemented in 12 rural health centres in Amhara, Ethiopia. The study enrolled 2399 pregnant women who were randomised to receive routine care, an enhanced nutrition package (iron and folic acid, monthly household supply of iodised salt, and micronutrient-fortified balanced energy protein supplement for undernourished women), an enhanced infection management package (genitourinary tract infection screening and treatment, and enhanced deworming), or both packages. In the present Longitudinal Infant Development and Growth study, a subset of 480 children of mothers from ENAT will be recruited equally from each of the four study arms and visited at 12, 18, and 24 months of postnatal age. We will evaluate a range of domains and deploy multiple measures to assess child neurodevelopment, including resting electroencephalography and visual evoked potentials, Hammersmith Infant Neurological Examination, eye-tracking, Bayley Scales of Infant and Toddler Development (Bayley-III), and Magnetic Resonance Imaging (MRI).

**Discussion:**

This study will advance understanding of the impact of nutrition and inflammation in pregnancy on long-term offspring neurodevelopment. This study aims to fill a critical knowledge gap on the benefits of prenatal interventions to promote the health of mothers and their offspring.

**Ethics and dissemination:**

This study was approved by the Institutional Review Boards of Addis Continental Institute of Public Health (ACIPH/IRB/002/2022) and Mass General Brigham (2023P000461). Results will be disseminated to local and international stakeholders.

**Trial registration number:**

NCT06296238.

**What is already known on this topic**

- In low- and middle-income countries, undernutrition and infections are prevalent risk factors for adverse child health and developmental outcomes.
- Undernutrition and exposure to inflammation in early infancy may adversely impact child neurodevelopment.

**What this study hopes to add**

- Increase evidence on the independent and combined effects of prenatal nutrition and infection interventions (ie, in utero exposures) on long-term offspring neurodevelopment in rural Ethiopia.
- Evaluate the implementation of advanced neuroimaging technologies to objectively assess neurodevelopment in young children in a rural research site in Ethiopia.

**How this study might affect research, practice, or policy**

- This study aims to fill a critical knowledge gap on the benefits of prenatal interventions and combining nutrition and health interventions in pregnancy to promote the long-term developmental potential of children worldwide.

## Introduction

 Approximately 200 million children under the age of 5 years worldwide do not reach their full developmental potential, most of whom are living in low- and middle-income countries (LMICs).^1^ The ‘first 1000 days’ represent a critical window of opportunity to optimise the developmental potential of children given rapid neurological and cognitive maturation during this timespan.^2^ During the late foetal and early neonatal period (~24 to 44 weeks gestation), the brain undergoes the most rapid growth and development, characterised by an increase in weight, new functional connections, and rapid gyrification.^3 4^ Identifying effective strategies in the prenatal and neonatal period is a key priority to optimise early childhood development globally.

Undernutrition and inflammation are prevalent and modifiable pregnancy risk factors that may adversely impact brain development in utero. Key macronutrients, such as protein, fat, carbohydrates, and micronutrients are required for cell proliferation, differentiation, growth factor synthesis, myelination, and synaptogenesis.^4^ The rapidly developing foetal brain is also highly susceptible to the effects of inflammation.^5^ Specifically, maternal infections during pregnancy may adversely influence brain development through activation of the maternal immune response.^6 7^

### Parent pregnancy study

**Figure 1:**
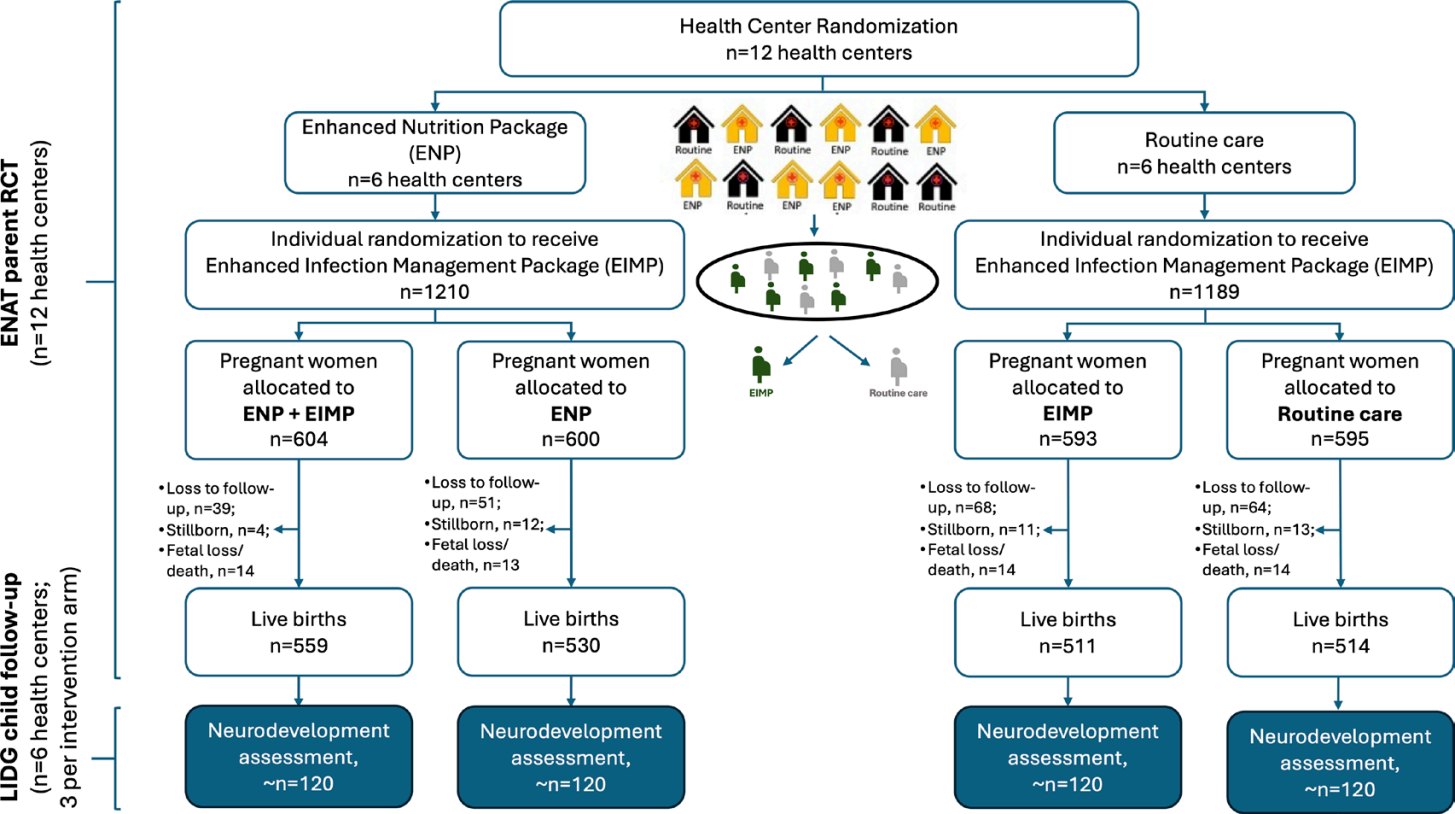
Design and participant flow of the ENAT parent RCT and LIDG child follow-up study. ENAT, Enhancing Nutrition and Antenatal Infection Treatment; LIDG, Longitudinal Infant Development and Growth; RCT, randomised controlled trial.

The Enhancing Nutrition and Antenatal Infection Treatment (‘ENAT’, mother in Amharic) study (ISRCTN15116516) was a pragmatic, open-label, 2×2 factorial, randomised clinical effectiveness study that tested the impact of packages of antenatal interventions to enhance maternal nutrition and manage pregnancy infections on birth outcomes in the rural Amhara region of Ethiopia.^8^ 12 rural health centres (clusters) were randomised to provide routine care or an enhanced nutrition package (ENP), including a micronutrient-fortified balanced energy protein supplement for undernourished women (mid-upper arm circumference (MUAC) of <23 cm), iron and folic acid, a regular supply of adequately iodised household salt and nutritional counselling (see figure 1). Pregnant women (n=2399) attending the study health centres were individually randomised to receive routine care or an enhanced infection management package (EIMP), which included enhanced deworming and screening and treatment of urinary tract infection, gonorrhoea/chlamydia, and symptomatic bacterial vaginosis or trichomonas. Birth weight and length were the primary outcomes of the pregnancy study.

### Infant-child follow-up study

**Figure 2:**
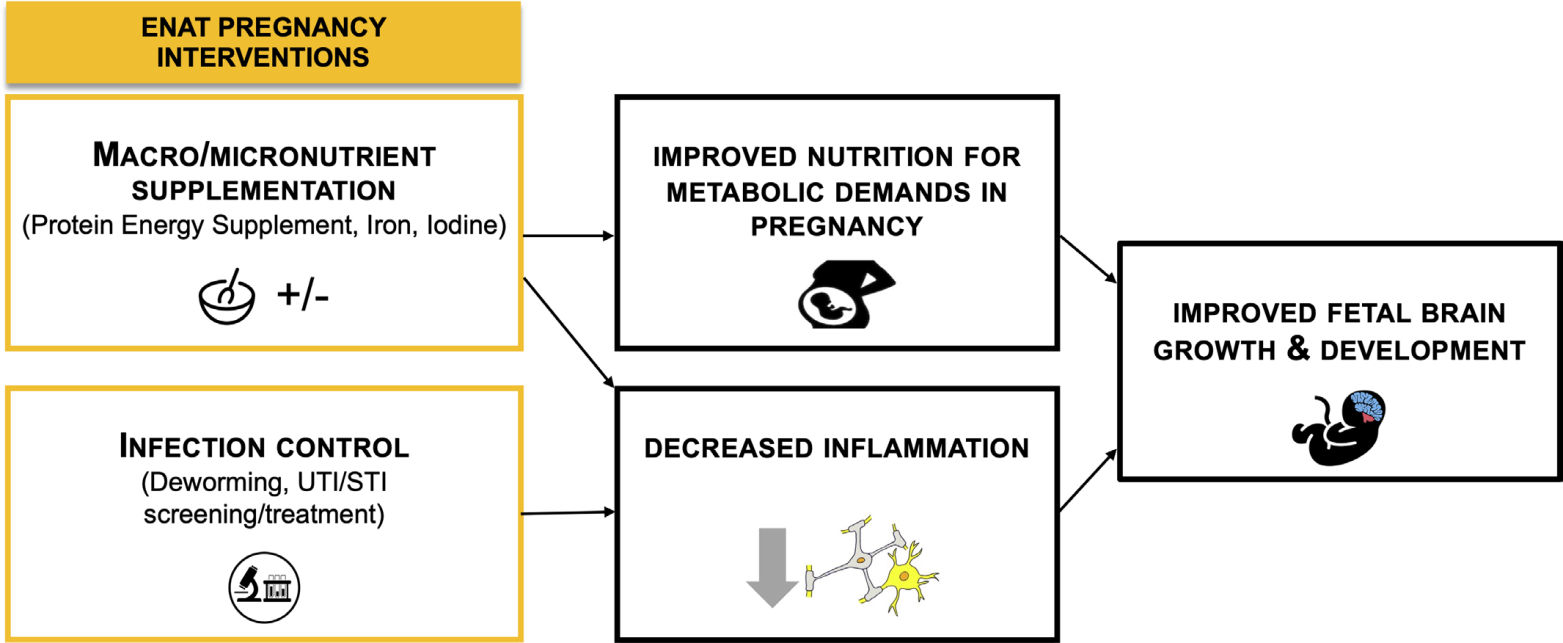
Concept diagram showing pathways of ENAT study interventions and effects on child neurodevelopment. ENAT, Enhancing Nutrition and Antenatal Infection Treatment.

In the current Longitudinal Infant Development and Growth (‘LIDG’, child in Amharic) study, we are conducting a follow-up of ENAT study offspring (n=480) until 24 months postnatal age. In addition to routine measures of global child development, this study will employ advanced and objective neurophysiological measures, including electroencephalography (EEG) and visual evoked potentials (VEP), eye-tracking, and neuroimaging (MRI) to assess child neurodevelopment. The overall goal of this study is to examine the effects of prenatal nutrition and infection management interventions on long-term child health, growth, and neurodevelopment. Figure 2 describes the pathways linking ENAT interventions to child growth and neurodevelopmental outcomes.

## Methods

### Study site

The parent ENAT study was implemented as a partnership with the Addis Continental Institute of Public Health (ACIPH), Amhara Regional Health Bureau (ARHB), Amhara Public Health Institute (APHI), and Brigham and Women’s Hospital (BWH)/Harvard Medical School. LIDG study participants were recruited from 6 of the 12 rural health centres involved in the parent study. The six centres participated in the biospecimen sub-studies of the ENAT pregnancy cohort and were chosen due to laboratory capacity. Three of the health centres were randomised to receive the ENP intervention and three were non-ENP health centres. A Hyperfine MRI machine is located at Felege Hiwot Referral Hospital, a regional referral hospital in Bahir Dar, Amhara.

### Study design

LIDG is an observational, longitudinal child follow-up study, following offspring of women who participated in the ENAT study.

### Patient and public involvement

Community involvement is crucial, especially when involving the use of technologies that are new to the community. During the initial study phase, we held a consultative meeting with representatives of the ARHB, APHI, and other relevant stakeholders to discuss the overall aim of the study and incorporate inputs to guide implementation plans. Formative research via key informant interviews was conducted during the design phase of the study with healthcare providers and community members, including mothers who participated in the parent ENAT study, fathers, in-laws, religious/community leaders, and other relevant stakeholders to develop a community-guided research strategy for introducing novel neurodevelopmental assessments, including a low-field MRI and a mobile, low-density EEG system. Key informants were identified during their visits to the study health centres by community health workers and the study team. Participants were interviewed about strategies to engage the community and they were also provided with the initial introductory videos to provide feedback on what information should be added to clarify the study procedures. These efforts informed the design and refinement of educational materials for study participants and the community sensitisation approach.

### Study participants and recruitment

Offspring of women in the ENAT parent study are recruited and enrolled for the LIDG study at the 12-month postnatal visit starting March 2023. Study participants are recruited during a visit to the study health centre, at home visits, or health posts. Field data collectors use introductory videos about MRI and EEG during community sensitisation and at enrolment to help mothers understand study procedures. As of May 2024, 462 children have been enrolled for follow-up assessments of health, growth, and development. Of these, 240 children have been consecutively recruited for advanced neurodevelopmental assessments using EEG and 60 with MRI, targeting children whose mothers had an MUAC <23 cm at the ENAT enrolment visit and/or received the balanced energy protein supplement to enhance the representation of children of undernourished mothers.^8^

Inclusion criteria include children with:

- Mothers who have participated in ENAT who consent to participate in the child follow-up LIDG study.
- Mothers who intend to stay in the study catchment area

Exclusion criteria for children include:

- Major congenital anomalies
- Severe morbidity or developmental disorder
- Acute symptoms of illness (eg, headache, vomiting, or dizziness).
- Neonatal encephalopathy

### Study procedures and data collection

Table 1 describes the timeline of study visits, assessments, and data collection. Children are followed at 12, 18, and 24 months of postnatal age (±3 months). For the 24-month visit, we conduct the assessments in the following order: eye-tracking, EEG, and Bayley-III assessment. Prior to the study initiation, the study procedures and order were piloted and tested among infants from the local study population to assess the feasibility and time required to complete the study visit. Modifications to study procedures and study protocols were made based on the findings and learning from the pretesting of study activities.

**Table 1:** Timeline of Longitudinal Infant Development and Growth study visits, assessments, and data collection

Assessment	Enrolment12 months (±3)	Visit 218 months (±3)	Visit 324 months (±3)
Neurodevelopment			
HINE	X		
Eye-tracking	X		X
Bayley-III			X
*Advanced neurodevelopmental assessments*			
EEG/VEP*			X
MRI†	X		
Child nutrition and growth			
Anthropometrics, diet quality questionnaires/survey	X	X	X
Maternal and child questionnaires			
Home environment	X	X	X
Child health history	X	X	X
Biospecimen			
Dried blood spot (child)	X		

* Participant subset, n=240.

† Participant subset, n=150.

Bayley-III, Bayley Scales of Infant and Toddler Development, Third Edition; EEG/VEP, electroencephalography with visual evoked potential; HINE, Hammersmith Infant Neurological ExaminationMRImagnetic resonance imaging

All data collection and study measurements are conducted by trained research staff (study nurses and field data collectors) at the study health centres, except MRI, which is conducted at the Felege Hiwot Referral Hospital. Research staff with expertise in MRI, EEG, and/or neurodevelopmental assessments provided in-depth training for each assessment modality in children of different ages to a cadre of physicians, radiology technicians, and study staff. Both in-person and virtual training sessions were conducted for up to 2 weeks to ensure competency in study procedures by study clinicians.

### Nutrition and growth

We collect interview-based data on breastfeeding, maternal diet, complementary feeding, food frequency, and household food security. Child anthropometrics are measured at each study visit using standard operating procedures, equipment and standardisation procedures as described by Intergrowth-21st.^9^ Child weight is measured using a high-quality digital infant scale (ADE M112600 Germany; precision 5 g) or standing scale (ADE M317600, precision 100 g). Child length is measured using a portable infantometer (Perspective Enterprises PE-RILB-LTWT, Michigan USA, precision 1 mm). Recumbent length is recorded to the last completed, and not the nearest, mm. For older children, standing height is measured using a stadiometer. Head, chest, and MUAC are measured to the nearest mm using insertion tapes (Shorr Productions, Maryland, USA). Regular calibration checks are made before each use of weighing scales, and length boards to ensure accuracy of measurement.^10 11^ Measurements are taken in duplicate at each study visit, with a third measure taken if the difference exceeds the pre-specified minimum detectable difference.

### Child health

We collect data on child health and morbidity, including signs of illness, hospitalisation, and vaccinations, through caregiver questionnaires and/or available medical records. Data on medical diagnoses, nutritional status, and treatments are extracted from medical records at the health centre of enrolment, if available.

### Home environment

We administer questionnaires to assess the child’s home environment, including measures of family socioeconomic status, daily hardships, family care indicators (UNICEF Multiple Indicator Cluster Surveys), and maternal mental health (Patient Health Questionnaire-9).^12^

## Neurodevelopmental assessments

### Hammersmith Infant Neurological Examination

The Hammersmith Infant Neurological Examination (HINE)^13^ is a standardised neurological assessment of infant tone, motor patterns, spontaneous movements, reflexes, visual-auditory attention, and behaviour. Study nurses were trained and standardised by paediatricians with expertise in neonatal neurological examination prior to the start of the study. Furthermore, a paediatrician-trainer provided refresher training to study nurses approximately 6 months after the initial training.

### Eye-tracking

Culturally adapted task videos designed to assess visual attention and recognition memory (a short Sesame Street video: *Cecile—Up Down In Out*^1^; and Multisensory Attention Assessment Protocol)^15^ are displayed on a tablet, while a secondary tablet records the child’s gaze behaviour. Recorded eye movements are analysed using the Online Webcam-Linked Eye Tracker^16^ software. Study nurses were trained on the eye-tracking protocol before data collection. Videos are checked regularly at the start of the study and periodically throughout the study to review the videos’ quality and the child’s positioning.

### Bayley-III

The Bayley-III has been validated in the sub-Saharan African setting to measure cognition, language, and motor development.^17 18^ Study nurses were trained on the administration of the Bayley-III by research team members certified in the administration of the tool. A 90% inter-observer reliability score between trainer and trainee is required for research staff to administer the Bayley-III and 10% of assessments are videotaped with caregiver consent for external quality control.

### Advanced neurodevelopmental assessment (sub-set of participants)

#### Resting EEG and visual evoked potential

Resting EEG and VEP (EEG/VEP) are recorded using a portable EEG system (Enobio-32, Neuroelectrics, Barcelona, Spain) that has been used in a large-scale, home-based infant study.^19^ For resting EEG, children watch a short video, akin to a screen saver, while they sit on their mother’s lap. Children watch a pattern-reversal checkerboard as part of the VEP paradigm. Data quality is routinely monitored by trained research staff at BWH and Boston Children’s Hospital through visual inspection of EEG recordings against a previously validated quality assessment checklist.^19^ Feedback is provided to study staff as necessary.

#### EEG processing

Resting neural oscillations and VEP waveforms will be extracted from the EEG recording using open-source EEG processing scripts that have been validated for infant EEG data collected in low-resource settings. Power spectral densities will be calculated across all segments for each participant. Absolute and relative power will be scaled in dB/Hz, that is, 10×log10 (power) to assess four widely studied frequency bands, and age-appropriate boundaries will be used to define them: theta (3–5 Hz), alpha (6–9 Hz), beta (10–20 Hz), gamma (21–40 Hz). Global EEG power will be calculated by averaging across all electrodes over all accepted segments. Consistent with another large-scale randomised controlled trial using mobile EEG,^19^ we will compare differences in absolute and relative power values between groups, as nuisance variables that commonly impact absolute power values (eg, skull thickness, hair, etc.) should be randomly distributed. We will also extract peak latencies, amplitudes, and component durations for the VEP from electrodes over the occipital midline. Analyses will focus on the peak amplitude and latency of the first positive component (P1).

#### MRI

MRI scans are performed at ~12 months using the Hyperfine 0.064 Tesla low-field MRI scanner. Children are fed, swaddled, and soothed to sleep before scanning, and noise-attenuating ear protection is provided.^20 21^ T1 and T2 axial, coronal, and sagittal sequences are obtained (30 min). MRI analyses will include 2D brain metrics (bifrontal, biparietal, and transverse cerebellar diameters) and 3D regional and whole brain volumetry. If any clinical concerns are identified, a study-affiliated physician will be responsible for discussing the findings with the family and assisting with appropriate management and referrals. All abnormal scans and a random 10% of all scans will be reviewed externally by an external MRI expert for quality control.

#### Biospecimens

At the enrolment/12-month visit, a haemoglobin level is determined and a blood sample (heel stick/finger prick) is collected on filter paper (Whatman card) for future analysis.

#### Incidental findings

In the event of any incidental findings during the course of the study, a physician affiliated with the study discusses the results with the family and arranges appropriate follow-up with a local physician and/or specialist. Study staff and the consulting paediatrician facilitate linkage and referral for the study participant and support costs associated with patient referral.

### Data management

The core of the data collection system is the Survey Solutions platform (The World Bank Group). Study nurses enter data directly into electronic tablets with programmed validity checks during study visits. Paper forms are used if tablets are temporarily unavailable due to power outage. A web-based dashboard supports data collectors, supervisors, and investigators in real-time management and monitoring of study activities.

### Laboratory analyses

#### Urinary iodine concentration

We will determine maternal iodine and creatinine concentrations using previously collected urine samples from the parent ENAT study, in which random spot urine samples (~20 mL) were collected. Urinary iodine concentration (UIC) of each sample will be analysed in duplicate using Pino modification of the Sandell-Kolthoff reaction method with spectrophotometric detection at the Ethiopian Public Health Institute (Addis Ababa, Ethiopia). The intra- and inter-assay coefficients of variation for the UICs will be calculated to assess the reliability of the test.

#### Thyroid function

Maternal dried blood spot samples were collected in the parent ENAT study and will be analysed at the University Children’s Hospital Zurich (Zurich, Switzerland) for concentrations of thyroglobulin,^22^ thyroid stimulating hormone and total thyroxine (T_4_) using ELISA assay methods adapted for dried blood spots.

#### Iron and inflammation biomarkers

In the parent ENAT study, an early morning maternal fasting blood sample was collected from participants at enrolment (gestational age≤24 weeks) and in the third trimester. Umbilical cord blood was also collected at delivery. We will measure serum ferritin and soluble transferrin receptor in maternal serum, which will be adjusted for inflammation (C-reactive protein (CRP) and alpha-1-acid glycoprotein (AGP)) as recommended by WHO and Biomarkers Reflecting Inflammation and Nutritional Determinants of Anemia.^23^,^25^ Serum hepcidin will be measured using validated competitive ELISA.^26^ Interleukin (IL)-6, IL-6R, CRP, and AGP will be analysed using validated ELISA and electrochemiluminescence multiplex platforms (Meso Scale Discovery Sector Imager 2400, Gaithersburg, Maryland).

### Study outcomes

The primary and secondary outcomes of the LIDG study are described in table 2.

**Table 2:** Primary and secondary outcomes of the Longitudinal Infant Development and Growth study

Primary outcome(s)	
P1. Resting brain function	Absolute power of alpha frequency band on mobile electroencephalography
Secondary outcome(s)	
S1. Resting brain function	Absolute power of beta, theta, gamma frequency bands on mobile electroencephalography
S2. Neural processing speed	Visual evoked potential (VEP) P1 Latency
S3. Visual attention	Measured with infant eye tracking during *Cecile—Up-Down-In-Out* Attention Task; Multisensory Attention Assessment Protocol
S4. Hammersmith Infant Neurological Examination at 12 months	Standardised neurobehavioural examination that includes 26 signs assessing infant cranial nerve function, movement, reflexes, behaviour, and gross and fine motor function
S5. Cognitive development at 24 months	Bayley Scales of Infant and Toddler Development (Third Edition), Cognition Score
S6. Head circumference at 24 months	Occipito-frontal head circumference
Exploratory outcome(s)	
E1. Resting brain function	Relative power of alpha, beta, theta, gamma frequency bands on mobile electroencephalography
E2. Functional brain connectivity	Neural network efficiency and organisation across brain regions measured by mobile electroencephalography

### Statistical analysis

Descriptive statistics of baseline demographic and clinical characteristics will be reported overall and by study arm at both the cluster and individual level.^27^ We will examine loss to follow-up in each study and the characteristics related to it. Using intention-to-treat principles, we will determine marginal intervention effects (ENP vs non-ENP, EIMP vs non-EIMP), and the combined effect of ENP+EIMP package compared with no intervention. The primary comparison is the ENP versus non-ENP effect. To estimate marginal ENP effects (cluster-randomised) on neurodevelopmental outcomes, given the small cluster number we will use cluster-level analysis^28^ to compare cluster means between nutrition study arms using the t-test. Mean covariate-adjusted outcomes for each cluster will be calculated by linear regression. To estimate marginal EIMP effects (individually randomised), we will use generalised estimating equations with robust variance to account for health centre clustering. In analyses of intervention effects, we will adjust for a priori prognostic factors of outcome (maternal age, parity, education, wealth, household size) and imbalanced covariates. To examine the intervention impacts on biochemical biomarkers, we will use a similar approach as described above, applying non-parametric methods as required. We will also determine the associations between maternal third trimester biochemical biomarker status (thyroid function, inflammation, and iron status) with primary and secondary outcomes using mixed linear regression models, adjusted and unadjusted for a priori*-*defined potential confounders. We assess intervention effects on several secondary outcomes at different study visits and time points. Each individual secondary outcome (S2–S6) will be interpreted based on individual null hypothesis, and will not be adjusted for multiplicity.^29^ For the secondary EEG outcomes (S1) we will present results with and without adjustment for multiple testing using Hochberg method.^30^ For exploratory EEG outcomes (E1, E2), we will present results both with and without adjustment for multiple testing using False Discovery Rate.^31^

### Sample size

We estimated the minimum detectable effect size for the marginal effects of each package (ENP vs non-ENP or EIMP vs non-EIMP). For the nutrition package, we accounted for the design effect due to clustering from six health centres, (k=6) and assumed a two-sided test with α=0.05 and power of 80%.^32^ For the primary outcome (absolute power of alpha band on EEG), with an estimated 200 EEGs of adequate quality and an inter-cluster correlation (⍴)=0.01 (cluster sample size, m=33; design effect (D)=1+(33−1)×0.01=1.32), we will have 80% power to detect a difference between nutrition study arms (ENP vs non-ENP) of moderate effect size (Cohen’s d=0.46). For the infection package (individually randomised), the minimum effect size for the EIMP effect (vs non-EIMP) is d=0.40.

For the secondary outcome (Bayley-III), a sample of 400 children assessed at 24 months (200 per arm for marginal ENP effects assuming ~17% loss to follow-up of the original n=480; m=67; D=1.66) is sufficiently powered to detect differences between nutrition study arms of small-to-moderate effect size of d=0.37. The minimum effect size for the infection arm effect (individually randomised) is d=0.28. Previous nutritional intervention studies have reported effect sizes at or above this level, for example, Bayley-III 12-month cognitive function (d=0.40) and Bayley-II 12-month psychomotor developmental index (d=0.51), orientation-engagement percentile (d=0.45), and motor quality (d=0.74).^33^

### Ethics and dissemination

The study is approved at Partners Healthcare (Mass General Brigham; 2023P000461) and ACIPH (ACIPH/IRB/002-A1/2022) Institutional Review Boards.

### Confidentiality

Data collected for this study are kept strictly confidential at ACIPH on a local encrypted server. Personal identifiers were not used in study-specific forms, aside from the identifier module. Paper copies of data forms for data entry and analysis were stored in a locked file when not in use. Access to data files containing personal identifying information is limited to the principal investigators and key staff.

### Dissemination plan

Findings of the LIDG study will be disseminated to the Ethiopian Ministry of Health, ARHB, APHI, zonal health departments, woreda health offices, community representatives from each of the study sites, and other relevant stakeholders. In collaboration with the healthcare providers at the study sites, we will also organise workshops at the study health centres to disseminate the study findings to parents of children involved in the study. The engagement of relevant stakeholders in the dissemination process is expected to enhance ownership of the research output and facilitate integration of the findings into local programmes. Findings will be disseminated as presentations in workshops, symposiums, and conferences at local, regional, national, and international levels as appropriate; as well as reports and peer-reviewed journal publications.

## Discussion

The LIDG study examines the impact of targeted antenatal nutrition and infection management interventions during pregnancy on long-term child neurodevelopment using a novel battery of assessments, including MRI and EEG, in Ethiopia. This study is novel in its follow-up of infants born into a rigorous intervention study up to 24 months of age. However, there are limitations to consider. Due to resource constraints, the number of nutrition intervention clusters in the study are relatively small for the infant follow-up study (n=6). Follow-up and retention may be influenced by ongoing security concerns at the LIDG study site in Amhara, Ethiopia. Further, the follow-up period of 24 months postnatal, while critical for assessing early childhood development, may not be sufficient to capture the longer-term effects of prenatal interventions on cognitive, behavioural, and educational outcomes. Despite these challenges, the LIDG study will contribute towards the body of knowledge surrounding the complex interplay of nutrition, infection, and early childhood neurodevelopment in LMICs. The findings have the potential to inform maternal and child health policies and interventions on both regional and global scales.

## Data Availability

No data are available.
