# Supplementary material for: Impact of maternal antenatal nutrition and infection treatment interventions on Longitudinal Infant Development and Growth in rural Ethiopia: protocol of the LIDG child follow-up study
Source: BMJ Paediatr Open. 2024 Dec 24;8(1):e002840. doi: 10.1136/bmjpo-2024-002840 (PMC11683997; doi:10.1136/bmjpo-2024-002840)
Supplement: Supplementary file 1 [file bmjpo-2024-002840.reviewer_comments.pdf]

PEER REVIEW HISTORY

BMJ Paediatrics Open publishes all reviews undertaken for accepted manuscripts. Reviewers are asked to complete a checklist review form and are provided with free text boxes to elaborate on their assessment. These free text comments are reproduced below.

ARTICLE DETAILS

**TITLE (PROVISIONAL)**	Impact of Maternal Antenatal Nutrition and Infection Treatment Interventions on Longitudinal Infant Development and Growth (LIDG) in Rural Ethiopia: Protocol of a Child Follow-Up Study
**AUTHORS**	Abate, Firehiwot Workneh; Chin, Theresa I.; Yibeltal, Kalkidan; Fasil, Nebiyou; North, Krysten; Jensen, Sarah K. G.; Kidane, Workagegnehu Tarekegn; Melese, Mulatu; Tsegaye, Sitota; Berhane, Yoseph Yemane; Roy Paladhi, Unmesha; Abate, Betelhem Haimanot; Teklehaimanot, Atsede; Melka, Tizita Lemma; Pihl, Stephen; An, Winko W.; Van Dyk, Fred; Mullany, Luke C.; Folger, Lian V.; Cherkerzian, Sara; Troller-Renfree, Sonya V.; Thomason, Moriah E.; Andersson, Maria; Inder, Terrie; Nelson, Charles A.; Grant, P. Ellen; Christian, Parul; Worku, Alemayehu; Berhane, Yemane; Lee, Anne

VERSION 1 - REVIEW

**REVIEWER NAME**	***Samudragupta Bora***
**REVIEWER AFFILIATION**	University Hospitals Rainbow Babies & Children's Hospital, Pediatrics
**REVIEWER CONFLICT OF INTEREST**	
**DATE REVIEW RETURNED**	13-Aug-2024

**GENERAL COMMENTS**	The current study protocol is for the neurodevelopmental follow-up until 24 months of age of a subset of children (n=480) born to mothers (n=2,399) randomized to receive routine care, an enhanced nutrition package, an enhanced infection management package, or both packages as part of a pragmatic, open-label, 2x2 factorial, randomized effectiveness study in 12 rural health centers in Amhara, Ethiopia. The longitudinal design, a comprehensive battery of neurodevelopmental assessments, and appropriate statistical power to identify meaningful differences between study arms are among the strengths of this follow-up study. Most importantly, the study is guided by a strong conceptual framework and targets an important clinical and public health issue. A few minor recommendations to improve the quality of the study and protocol reporting are described below.<br>1. The current protocol does not specify whether the parent study results are available. If they are available, please summarize their findings in the background section.<br>2. The study's comprehensive neurodevelopmental assessment battery is a strength, but it may be time-consuming and effort-intensive for participants and their families, given the study settings. It would be helpful to know whether the entire 24-month battery has been pilot-tested at the study sites. It would also be useful to understand the order in which different assessments will be administered. I strongly recommend that the authors include a

	section that details the pilot process and how the pilot data shaped the current protocol.<br>3. The proposed neurodevelopmental assessment battery is extremely complex for resource-constrained settings, relying heavily on the expertise and competency of multidisciplinary assessors. I strongly recommend that the authors describe in detail the training and quality control measures and processes that will be implemented.<br>4. It is unclear why children with severe morbidity or developmental disorders, as well as acute illness symptoms such as headache, vomiting, or dizziness, will be excluded.<br>5. The study protocol fails to adequately describe the section on patient and public involvement. Given the complexities of the study processes, I propose that this section be revised to include specific information about the follow-up study, such as who was consulted, how many individual stakeholders were consulted, how they were recruited, who conducted the interviews, and so on. Unfortunately, the current information is overly generic and offers little value concerning the follow-up study.<br>6. Additional information is required for the MRI protocol, which will be carried out on only 150 children from the current study subset with a 0.064 Tesla low-field MRI scanner. It is unclear whether the scans will have sufficient spatial resolution to score brain abnormalities using the Kidokoro classification system.<br>7. While the child battery is extremely comprehensive, the proposed maternal/parental assessment battery appears to be limited in scope. Furthermore, the reason for including PHQ-9 is unclear.<br>8. The dissemination plan fails to adequately describe how the study's findings will be communicated to the parents and families involved. The approach tailored to the study setting must be clearly described because it is an important component of the study protocol.<br>9. The protocol should also include the reporting of outcomes and referral practices for children identified with neurodevelopmental impairments during the follow-up study.<br>10. Finally, I strongly recommend a limitations section in the protocol.

**REVIEWER NAME**	***Jon Dorling***
**REVIEWER AFFILIATION**	University Hospital Southampton NHS Foundation Trust, Department of Neonatal Medicine
**REVIEWER CONFLICT OF INTEREST**	
**DATE REVIEW RETURNED**	16-Sep-2024

**GENERAL COMMENTS**	This is an interesting study that is well described in this protocol paper that can be published with a few very minor amendments<br><br>line 52 "iron folic acid" is presumably missing a comma (i.e. "iron, folic acid")<br><br>line 85 "This study will fill a critical knowledge gap on the benefits of prenatal interventions" is rather presumptuous as it may not be successful if the results are equivocal. I would suggest "This study aims to fill a critical knowledge gap on the benefits of prenatal interventions". The same issue is present in line 63

	line 275. The authors should describe their approach to the use of multiple outcomes. Will they be adjusting for multiple hypothesis tests (e.g. using a Bonferroni correction or similar?

VERSION 1 – AUTHOR RESPONSE

Reviewer: 1

Dr. Samudragupta Bora, University Hospitals Rainbow Babies & Children's Hospital, The University of Queensland

Comments to the Author

The current study protocol is for the neurodevelopmental follow-up until 24 months of age of a subset of children (n=480) born to mothers (n=2,399) randomized to receive routine care, an enhanced nutrition package, an enhanced infection management package, or both packages as part of a pragmatic, open-label, 2x2 factorial, randomized effectiveness study in 12 rural health centers in Amhara, Ethiopia. The longitudinal design, a comprehensive battery of neurodevelopmental assessments, and appropriate statistical power to identify meaningful differences between study arms are among the strengths of this follow-up study. Most importantly, the study is guided by a strong conceptual framework and targets an important clinical and public health issue. A few minor recommendations to improve the quality of the study and protocol reporting are described below.

1. The current protocol does not specify whether the parent study results are available. If they are available, please summarize their findings in the background section.

Authors Response: Results from the parent study are not yet published. If they are published prior to publication of this article we will include the primary trial results.

2. The study's comprehensive neurodevelopmental assessment battery is a strength, but it may be time-consuming and effort-intensive for participants and their families, given the study settings. It would be helpful to know whether the entire 24-month battery has been pilot-tested at the study sites. It would also be useful to understand the order in which different assessments will be administered. I strongly recommend that the authors include a section that details the pilot process and how the pilot data shaped the current protocol.

Authors Response: All assessments are pilot-tested for feasibility prior to study implementation. We agree with the reviewer regarding the extensive nature of the 24-month visit, which consists of multiple child assessments, and have added details pertaining to the pilot testing procedures and order of assessments as recommended (see Lines 182-187).

3. The proposed neurodevelopmental assessment battery is extremely complex for resource-constrained settings, relying heavily on the expertise and competency of multidisciplinary assessors. I strongly recommend that the authors describe in detail the training and quality control measures and processes that will be implemented.

Authors Response: The study authors recognise the importance of rigorous training and quality control measures, particularly for the complex study design proposed. Prior to study implementation, extensive training was conducted for all study components, and are further detailed in the ‘Study

Procedures' section (Lines 190-194). Details for quality control measures specific to each neurodevelopmental assessment can be found as follows: HINE (see Lines 224-227), Eye-tracking (Lines 232-234), Bayley-III (Lines 237-240), EEG/VEP (Lines 246-249), MRI (Lines 267-271).

4. It is unclear why children with severe morbidity or developmental disorders, as well as acute illness symptoms such as headache, vomiting, or dizziness, will be excluded.

Authors Response: We have excluded children with severe morbidity or developmental disorders, and those with any acute illnesses for several reasons. Firstly, the current research aims focus on examining typical neurodevelopment among healthy children. Inclusion of those with severe morbidity or developmental disorders as well as those with acute symptoms may introduce confounding impacts of these conditions on outcomes measured. Additionally, the exclusion of these participants may help avoid the misattribution of the presence of any acute findings (e.g. headaches, dizziness) to study procedures. Finally, due to ongoing security concerns in Ethiopia, the feasibility of performing real-time data monitoring is significantly limited. If clinical findings emerge from the assessments (e.g. MRI), referral to health care providers would be delayed. This is of particular concern for those with severe morbidities requiring urgent care.

5. The study protocol fails to adequately describe the section on patient and public involvement. Given the complexities of the study processes, I propose that this section be revised to include specific information about the follow-up study, such as who was consulted, how many individual stakeholders were consulted, how they were recruited, who conducted the interviews, and so on. Unfortunately, the current information is overly generic and offers little value concerning the follow-up study.

Authors Response: We have amended this section to include further details as suggested by the reviewer (see 'Patient and Public Involvement', Lines 146-160).

6. Additional information is required for the MRI protocol, which will be carried out on only 150 children from the current study subset with a 0.064 Tesla low-field MRI scanner. It is unclear whether the scans will have sufficient spatial resolution to score brain abnormalities using the Kidokoro classification system.

Authors Response: We note the reviewer's comments and have amended the MRI data analysis plan accordingly (Line 267-271).

7. While the child battery is extremely comprehensive, the proposed maternal/parental assessment battery appears to be limited in scope. Furthermore, the reason for including PHQ-9 is unclear.

Authors Response: Whilst the authors recognise the importance of parental factors predicting long-term child neurodevelopment, several reasons limit the inclusion of more comprehensive parental assessments. During pilot testing, the parent questionnaire was determined to be too lengthy and of burden to participants. In lieu of this, the current parental assessments were further refined and selected in consultation with a team of co-investigators and child development experts from Boston Children's Hospital and Columbia University. One of the parental assessments included is maternal mental mental health, measured using the Patient Health Questionnaire-9 (PHQ-9). The PHQ-9 was selected due to its strong psychometric properties and established use in low-and-middle-income

countries , including Ethiopia (Habtamu et al. 2022, BMC Prim Care; Degefa et al. 2020, BMC Psychiatry; Woldetensay et al. 2018, PLOS One). Further, upon pilot-testing the alternative use of the Edinburgh Postnatal Depression Scale, we found that its translation to Amharic was not favourable.

8. The dissemination plan fails to adequately describe how the study's findings will be communicated to the parents and families involved. The approach tailored to the study setting must be clearly described because it is an important component of the study protocol.

Authors Response: We have included further information regarding the dissemination of findings to parents and families involved (see ‘Dissemination Plan’, Lines 362-366).

9. The protocol should also include the reporting of outcomes and referral practices for children identified with neurodevelopmental impairments during the follow-up study.

Authors Response: We have included further information regarding referral practices in the event of any incidental findings or clinical concerns (see Lines 274-278).

10. Finally, I strongly recommend a limitations section in the protocol.

Authors Response: We have included a ‘Discussions’ section to summarise limitations of the LIDG study as recommended by the reviewer (see Lines 372-385).

Reviewer: 2

Prof. Jon Dorling, University Hospital Southampton NHS Foundation Trust

Comments to the Author

This is an interesting study that is well described in this protocol paper that can be published with a few very minor amendments

line 52 "iron folic acid" is presumably missing a comma (i.e. "iron, folic acid")

Authors Response: We have made revisions (i.e. “iron folic acid” to “iron and folic acid (IFA)”) accordingly (see Lines 51 and 115).

line 85 "This study will fill a critical knowledge gap on the benefits of prenatal interventions" is rather presumptuous as it may not be successful if the results are equivocal. I would suggest "This study aims to fill a critical knowledge gap on the benefits of prenatal interventions". The same issue is present in line 63

Authors Response: We have made revisions as suggested by the reviewer accordingly. (see Lines 62 and 84).

line 275. The authors should describe their approach to the use of multiple outcomes. Will they be adjusting for multiple hypothesis tests (e.g. using a Bonferroni correction or similar?

Authors Response: We have added further information on multiple comparisons in the Statistical Analysis section (see Lines 327-333).

VERSION 2 – REVIEW

**REVIEWER NAME**	***Samudragupta Bora***
**REVIEWER AFFILIATION**	University Hospitals Rainbow Babies & Children's Hospital, Pediatrics
**REVIEWER CONFLICT OF INTEREST**	
**DATE REVIEW RETURNED**	16-Nov-2024

**GENERAL COMMENTS**	Thank you for responding satisfactorily to all of the reviewers' comments. This is a valuable study, and I look forward to reading the results once it is completed. Best wishes.

**REVIEWER NAME**	***Shanti Raman***
**REVIEWER AFFILIATION**	South Western Sydney Local Health District, Department of Community Paediatrics
**REVIEWER CONFLICT OF INTEREST**	
**DATE REVIEW RETURNED**	15-Nov-2024

**GENERAL COMMENTS**	As the previous reviewer raised, I remain concerned about the battery of assessments and tests being undertaken on typically developing children in a low resource setting- including electroencephalography (EEG), visual evoked potentials (VEP), eye tracking and MRI. However, the research team has high level backing and have justified these tests- perhaps the residual capacity of the research team to impart knowledge and training on local staff is reasonable justification.
